# SARS-CoV-2 Genome Sequences, Including One with an ORF7a Deletion, Obtained from Patients in Bangladesh

**DOI:** 10.1128/MRA.00764-21

**Published:** 2021-12-09

**Authors:** Mizanur Rahman, Rummana Rahim, Abu Hasan, Naoko Kawai, Lavel Chinyama Moonga, Tatsuki Sugi, Kyoko Hayashida, Junya Yamagishi

**Affiliations:** a Evercare Hospital Dhaka, Dhaka, Bangladesh; b Division of Collaboration and Education, International Institute for Zoonosis Control, Hokkaido University, Sapporo, Japan; c International Collaboration Unit, International Institute for Zoonosis Control, Hokkaido University, Sapporo, Japan; Queens College CUNY

## Abstract

Genomic sequences from a complete SARS-CoV-2 open reading frame (ORF) were obtained from 24 patients diagnosed in May 2020 in Dhaka, Bangladesh. All sequences belonged to clade 20A or 20B, and none were variants of concern. Interestingly, one sequence showed a 161-nucleotide deletion in ORF7a.

## ANNOUNCEMENT

We sequenced a complete SARS-CoV-2 open reading frame (ORF) from 24 samples obtained in May 2020 in Dhaka. The study was approved by the Evercare Hospital Dhaka and Hokkaido University ethical committees (ERC 26/2020-2 and Jinjyu 2020-10, respectively).

Nasopharyngeal specimens with high threshold cycle (*C_T_*) values after reverse transcription-quantitative PCR (qRT-PCR) (Sansure Biotech, China) were used for the sequencing. RNA was purified using the MagMAX viral/pathogen nucleic isolation kit (Thermo Fisher Scientific). The ProtoScript II first-strand cDNA synthesis kit (E6560S; NEB) was used to synthesize cDNAs with oligo(dT) primers. Multiplex PCRs were conducted, using cDNAs and the ARTIC v1 primer set according to the published protocol, with 5′-CATTATGATCTTGCAGTTCAAGTGAG-3′ as the primer instead of the nCoV-2019_91_RIGHT primer. MinION sequencing was performed using flow cells (FLO-MIN106). Libraries were constructed using ligation sequencing (LSK-109) and native barcoding expansion (EXP-NBD103 and EXP-NBD114; ONT) kits following the manual’s instructions. Another multiplex PCR was conducted for MiSeq sequencing using TaKaRa Ex Taq hot-start (HS) DNA polymerase (RR006A) with three combinations of the same primers (Table 1) and the following conditions: 1× reaction buffer, 0.2 mM deoxynucleoside triphosphates (dNTPs), 1.44 μM primer mixture, and 0.625 units of Ex Taq in 25 μL reaction volume incubated at 98°C for 30 s, followed by 35 cycles of 98°C for 15 s and 65°C for 5 min. The amplicons of each sample were pooled, end-repaired using the NEBNext Ultra II end-repair/dA-tailing module (NEB), and purified using AMPure XP beads (Beckman Coulter). Afterward, the in-house Y-shaped adapter, which is comparable to that from the NEBNext Ultra II DNA library prep kit for Illumina (NEB), was ligated using blunt/TA ligase master mix (NEB). Another PCR added index sequences, and MiSeq 300-bp paired-end sequencing was performed using the MiSeq reagent kit v3 (Illumina).

**TABLE 1:** Primer sets for MiSeq sequencing^*a*^

pool_1		pool_2		pool_3	
nCoV-2019_1_LEFT	nCoV-2019_2_RIGHT	nCoV-2019_2_LEFT	nCoV-2019_3_RIGHT	nCoV-2019_3_LEFT	nCoV-2019_4_RIGHT
nCoV-2019_4_LEFT	nCoV-2019_5_RIGHT	nCoV-2019_5_LEFT	nCoV-2019_6_RIGHT	nCoV-2019_6_LEFT	nCoV-2019_7_RIGHT
…	…	…	…	…	…
nCoV-2019_94_LEFT	nCoV-2019_95_RIGHT	nCoV-2019_95_LEFT	nCoV-2019_96_RIGHT	nCoV-2019_96_LEFT	nCoV-2019_97_RIGHT
nCoV-2019_97_LEFT	nCoV-2019_98_RIGHT	nCoV-2019_98_LEFT	nCoV-2019_98_RIGHT	nCoV-2019_1_LEFT	nCoV-2019_1_RIGHT

a …, Primers from nCoV-2019_5_LEFT to nCoV-2019_93_LEFT and from nCoV-2019_8_RIGHT to nCoV-2019_94_RIGHT in order.

A total of 9.8 million paired-end reads were obtained with MiSeq sequencing. They were trimmed using pTrimmer v1.3.3 (1) and Trimmomatic v0.36 (2) and aligned using minimap2 v2.17 (3) with the reference sequence under GenBank accession number MN908947.3 (4). Sequence variants were called using GATK HaplotypeCaller v4.2.0.0 (5). Then, original genome sequences were reconstituted using GATK FastaAlternateReferenceMaker v4.2.0.0. With MinION sequencing, 52.1 million reads with a median of 509 bp were obtained. Regions with less than 5× coverage were masked with N bases or filled with MinION reads, obtained using Guppy_Basecaller v4.4.2 (ONT), when possible. Default parameters were used for all software unless otherwise specified.

The sequences were analyzed and assigned to clades using the Nextclade Web service (6). The phylogenetic analysis of 1,139 sequences originating from Bangladesh available in the Global Initiative on Sharing Avian Influenza Data (GISAID) database in June 2021, with a guide reference tree provided by Nextclade, was conducted and visualized using Nextclade. We observed one large deletion in ORF7a (hCoV-19/Bangladesh/ECHD_00521/2020, EPI_ISL_2350802). The region was amplified with primers, 5′-CGCTACTTGTGAGCTTTATCACTACC-3′ and 5′-CATTATGATCTTGCAGTTCAAGTGAG-3′, from the same cDNA as that subjected to next-generation sequencing and examined by Sanger sequencing using the same primers as those used for PCR. A 161-nucleotide (nt) frameshift deletion, from nt 27525 to 27685, was confirmed. The obtained genomes belonged to clades 20A (*n* = 8) and 20B (*n* = 16).

### Data availability.

The genome sequences are available from the GISAID and International Nucleotide Sequence Database Collaboration (INSDC) databases under accession numbers EPI_ISL_2350793 to EPI_ISL_2350816 and GenBank accession numbers LC647128 to LC647151, respectively. The raw sequence reads used in this study are available from the INSDC database under GenBank SRA accession numbers DRX308106 to DRX308153.
